# Elevated miR-103b in exosomes derived from brain-metastatic triple-negative breast cancer cells remodels the brain pre-metastatic niche (PMN)

**DOI:** 10.1080/15384047.2026.2725357

**Published:** 2026-09-25

**Authors:** Li Zhou, Jin Ke, Shanlan Zhang, Sufang Xiao, Hui Zhang, Shan Liu, Li Liu, Mingan Liu, Zuohua Lu, Songguo Zheng

**Affiliations:** a Department of Oncology, Songjiang Hospital Affiliated to Shanghai Jiao Tong University School of Medicine, Shanghai, China; b Department of Clinical Laboratory, Pudong Gongli Hospital, Shanghai University of Medicine & Health Sciences, Shanghai, China; c Department of Immunology, The School of Cell & Gene Therapy, Songjiang Research Institute and Songjiang Hospital Affiliated to Shanghai Jiao Tong University School of Medicine, Shanghai, China; d State Key Lab of Innovative Immune Therapy, Shanghai Jiao Tong University, Shanghai, China

**Keywords:** Triple-negative breast cancer, brain metastasis, exosomes, miR-103b, pre-metastatic niche, astrocytes, brain microvascular endothelial cells

## Abstract

**Background:**

Triple-negative breast cancer (TNBC) is a highly aggressive breast cancer with high brain metastatic (BM) potential. Tumor-derived exosomes are implicated as key modulators during the formation of the pre-metastatic niche (PMN). However, the regulation of TNBC-BM-derived exosomes on brain PMN remains enigmatic.

**Methods:**

The morphology and uptake of exosomes were identified using transmission electron microscopy and live-cell imaging, respectively. Differentially secreted miRNAs and mRNA were identified using high-throughput sequencing. The permeability of the blood‒brain barrier (BBB) and tight junction integrity were examined using immunofluorescence staining. Cell viability was examined using the CCK-8 assay. The apoptosis and intracellular ROS were investigated using fluorescence staining. The expression of mRNAs was examined using qPCR.

**Results:**

miR-103b was significantly increased in exosomes derived from TNBC-BM cells. miR-103b compromised the tight junctions of HUVECs and increased BBB permeability in mice. Additionally, miR-103b promoted apoptosis and increased intracellular ROS in HUVECs and U251 cells, two cell lines commonly used as surrogates for brain microvascular endothelial cells and astrocytes, respectively. miR-103b-regulated differentially expressed genes modulated tight junction, apoptosis, oxidative stress, inflammation, and metabolism. Target analysis identified 216 targets of miR-103b, among which hub targets were associated with biological processes involved in maintaining the BBB and the glucose metabolism signaling pathway.

**Conclusion:**

Collectively, our findings for the first time reveal that exosomal miR-103b from TNBC-BM cells potentially regulates the two major cellular constituents of the brain PMN, highlighting its role in the formation of the brain PMN during TNBC-BM and its potential as a promising therapeutic target.

## Introduction

Triple-negative breast cancer (TNBC) is a particularly aggressive subtype of breast cancer and shows a strong propensity for brain metastasis (BM).^1-3^ Patients diagnosed with brain metastasis from TNBC face a grim prognosis, with a median overall survival (OS) of less than 12 months.^4^ ^,^ ^5^ Currently, available therapeutic modalities for TNBC with BM (TNBC-BM) are still limited in improving OS.^6^ The development of effective targets for TNBC-BM continues to pose considerable challenges because its related molecular mechanism remains elusive.

Exosomes, secreted by various cells and as messengers in cell-to-cell communication, play a pivotal role in tumor pathology by regulating microenvironmental homeostasis through transferring cargo molecules from donor cells to neighboring or distant recipient cells.^7-11^ MicroRNAs, one of the major cargo molecules of exosomes, are established as key mediators of tumor progression and metastases owing to their ability to reprogram the phenotypic and functional characteristics of recipient cells at metastatic sites, thus fostering a pre-metastatic niche (PMN).^12-16^ Tumor-derived exosomal microRNAs are reportedly primary architects in establishing brain PMN through several behaviors, such as disrupting the BBB,^17-19^ regulating brain cell metabolism,^20^ and promoting the formation of an inflammatory microenvironment.^21^ Together, these studies highlight the functional diversity of tumor-derived exosomal microRNA in establishing brain PMN. However, the impact of TNBC-BM-derived exosomes on PMN remodeling remains poorly understood.

Here, we investigated the role of TNBC-BM-derived exosomal microRNA in modulating the brain PMN. First, we explored the differential expression of exosomal miRNA between the human TNBC cell line MDA-MB-231 and its brain metastatic variant MDA-MB-231Brm through high-throughput sequencing. Next, the effect of increased miR-103b in exosomes derived from MDA-MB-231Brm cells on the permeability of the BBB was investigated *in vitro* and *in vivo*. Considering that brain microvascular endothelial cells (BMECs) and astrocytes are two major cellular components of the PMN, we then examined the impact of miR-103b on HUMCs and U251 cells, two cell lines commonly used as surrogates for brain microvascular endothelial cells and astrocytes, respectively. These included analyzing changes in cell viability, apoptosis, and intracellular reactive oxygen species (ROS). Furthermore, we explored miR-103b-regulated mRNA profiles in HUMCs and U251 cells using high-throughput RNA sequencing. Gene Ontology (GO) enrichment, Kyoto Encyclopedia of Genes and Genomes (KEGG) pathway enrichment, inflammatory cytokine analysis, and target gene prediction were conducted to elucidate the biological processes, signaling pathways, inflammatory alterations, and targets regulated by miR-103b. Collectively, our findings provide new insight into the molecular mechanisms of TNBC-BM, whereby TNBC-BM-secreted exosomal miR-103b promotes the formation of brain PMN by remodeling the mRNA expression of vascular endothelial cells and astrocytes, highlighting its potential as a promising therapeutic target.

## Materials and methods

### Cell culture and transfection

The human TNBC cell line MDA-MB-231 was purchased from ATCC (catalog number: CRM-HTB-26), and its brain metastatic variant, MDA-MB-231Brm, was a kind gift from Professor Shao-jin You (Atlanta Education and Research Foundation, Atlanta VA Medical Center, Decatur, Georgia). The human umbilical vein endothelial cell lines HUVECs and human U251 astrocytic cells were purchased from the National Collection of Authenticated Cell Cultures (catalogue number of HUVECs: SCSP-5330; catalogue number of U251: TCHu 58). MDA-MB-231 and MDA-MB-231Brm cells were cultured in L-15 medium (Gibco, USA) supplemented with 10% heat-inactivated fetal bovine serum (FBS; Gibco, USA). HUVECs and U251 cells were cultured in DMEM high glucose medium (Gibco, USA) supplemented with 10% FBS. All the cells were grown in a humidified atmosphere with 5% CO_2_ at 37 °C. All the cell lines used in this study were authenticated before experimental use. Authentication was performed by short tandem repeat (STR) profiling at the National Collection of Authenticated Cell Cultures. The resulting STR profiles were compared to reference databases (ATCC) to confirm cell line identity. Authentication was repeated every six months or after ten passages, whichever came first. No misidentified or cross‑contaminated cell lines were detected.

For mycoplasma monitoring, cell cultures were routinely tested for mycoplasma contamination every two weeks and before any critical experiment. Testing was performed using the MycoAlert Mycoplasma detection kit (Lonza, USA). No mycoplasma infection was detected in any of the cell lines used during the course of the experiments.

For cell transfection, cells were seeded in a plate and grown to 60%–70% confluence. Then, the cells were transfected with miR-103b mimic (Syn-hsa-miR-103b miScript miRNA Mimic, GeneGlobe ID: MSY0007402, Qiagen, Germany) or its negative control (AllStars Negative Control miRNA, GeneGlobe ID: SI03650318, Qiagen, Germany) using Lipofectamine RNAiMAX reagent (Thermo Fisher Scientific, USA) according to the manufacturer’s instructions.

### Isolation of exosomes

To isolate exosomes, MDA-MB-231 cells and MDA-MB-231Brm cells were cultured in L-15 medium supplemented with 10% exosome-depleted FBS (by 100,000*g* ultracentrifugation) for 48 h. Then, the supernatant was harvested for exosome isolation. Exosomes in the supernatant were isolated using differential centrifugation as described previously.^22^ Briefly, the harvested supernatant was sequentially centrifuged at 2000 × g for 20 min, 4000 × g for 10 min, and 10,000 × g for 30 min to remove cells and cellular debris. Then, the resultant supernatant was transferred to a new ultracentrifuge tube and subjected to ultracentrifugation at 120,000 × g for 2 h in the SW32Ti rotor (Beckman Coulter, USA). After ultracentrifugation, the supernatant was removed, and the precipitate was resuspended in PBS and centrifuged at 120,000 × g for 2 h. Then, the collected precipitate containing the exosomes was resuspended in H_2_O for transmission electron microscopy (TEM) detection or resuspended in PBS for subsequent experiments. All isolation processes were performed at 4 °C.

### Transmission electron microscopy (TEM)

For the transmission electron microscopy assay, a 50 µL drop of the exosomal suspension was loaded onto a Formvar/carbon-coated copper grid and stained with 2% aqueous uranyl acetate for 2 min. Then, the stained exosome samples were examined using a Philips CM120 TEM (Philips Electron Optics, the Netherlands) at an acceleration voltage of 80 kV.

### Animals and lateral ventricle (ICV) injection of oligonucleotide

All the animal experiments were conducted under specific pathogen-free (SPF) conditions. The health status of the mice was continuously monitored throughout the study. The sentinel animals were housed within the same room and tested every four weeks for a comprehensive panel of murine pathogens, including but not limited to mouse hepatitis virus (MHV), minute virus of mice (MVM), Sendai virus, *Mycoplasma pulmonis*, *Helicobacter* spp., and endo/ectoparasites. All tests returned negative results during the entire experimental period. No known pathogens were detected in any of the mice used in this study.

Female C57BL/6J mice aged 8 weeks were purchased from Shanghai SLAC Laboratory Animal Co., Ltd. (Shanghai, China). All the mice were housed in individually ventilated cages (IVC), five mice per cage before surgery and one mouse per cage after surgery. All the mice were raised with standard laboratory conditions (room temperature: 22 °C ± 2 °C, relative humidity at 55% ± 5%, 12/12 h light/dark cycle, food and water ad libitum). All animal experiments were conducted in accordance with the European Communities Council Directive 2010/63/EU and the guidelines of the American Veterinary Medical Association (AVMA). All the animal experiments were approved by the Institutional Animal Care and Use Committee of Songjiang Hospital Affiliated to Shanghai Jiao Tong University School of Medicine (authorization number: ACE-001-2023).

A total of 24 female C57BL/6J mice were used. The sample size was determined by power analysis based on preliminary data. All the mice were randomly divided into 3 groups: the 5′-cy3-conjugated negative miRNA-3′cholesterol (5′-cy3-miR-negative-3′chol) injection group, the 3′cholesterol-conjugated 103b-miRNA mimics (miR-103b-3′chol) injection group, and the 3′cholesterol-conjugated negative miRNA control (miR-negative-3′chol) injection group, with 8 mice in each group. Randomization was used to allocate the animals to the control and treatment groups. The randomization sequence was generated using computer-generated random numbers in Excel. Specifically, the mice were assigned a sequential number upon arrival and then randomly allocated to groups using the randomization method described above. To minimize potential confounders, the order of treatments and measurements was randomized across the animals. All procedures (ICV injection and BBB permeability assessment) were performed at the same time of day to avoid circadian variation. The animals were housed in the same room under identical conditions (temperature, humidity, light/dark cycle), and the cage locations were rotated daily.

Before stereotaxic injection into the lateral ventricle, the mice were anesthetized using an isoflurane inhalation system. Anesthesia was induced with 4% isoflurane (Ningte®, Lunan Better Pharmaceutical Co., Ltd., Linyi, Shandong, China; National Drug Approval No. H20249627) in 100% medical-grade oxygen (Chinese Pharmacopoeia standard) and maintained with 1.5% isoflurane delivered via a nose cone. The depth of anesthesia was confirmed by the absence of a pedal withdrawal reflex. During the procedure, ophthalmic ointment was applied to protect the corneas, and body temperature was maintained at 37 °C using a heating pad.

For stereotaxic coordinates of the lateral ventricle, the mouse was mounted on a Kopf stereotactic device (Kopf Instruments). After scalp disinfection and skull exposure, the right lateral ventricle was marked according to the following coordinates: 0.2 mm posterior, 1.0 mm lateral to bregma, depth 2.2 mm, and a gauge was implanted. For miRNA injections, a volume of 5 μL of microRNA dissolved in saline was infused at a rate of 0.5 μL/min. Following the infusion, the needle was left in place for 10 min before being slowly withdrawn. The surgical wound was then disinfected and sutured. For postoperative analgesia, carprofen (5 mg/kg, subcutaneous injection) was administered immediately before the end of surgery and again 24 h later. For postoperative infection control, enrofloxacin (5 mg/kg, subcutaneous injection, once daily for three consecutive days) was administered. After surgery, the mice were placed in a recovery cage on a 37 °C heating pad and monitored until they were fully awake and ambulatory. All anesthetic and analgesic procedures were performed in accordance with the AVMA Guidelines for the Anesthesia and Analgesia of Animals (2020 Edition). Any animal showing severe adverse effects would have been immediately euthanized.

### FITC-dextran injection, perfusion, and tissue collection

Seven days after the ICV injection, FITC-dextran (2000 kDa) (FD2000S, Sigma Chemical Co., USA) was administered via the tail vein at a dose of 0.3 mg per gram of body weight. Two hours later, the mice were deeply anesthetized with an intraperitoneal injection of sodium pentobarbital (100 mg/kg). The depth of anesthesia was confirmed by the absence of a pedal withdrawal reflex. The mice were then subjected to transcardial perfusion sequentially with phosphate-buffered saline (PBS) followed by 4% paraformaldehyde (PFA). After perfusion, the brains were carefully harvested and post-fixed in 4% PFA at 4 °C.

### Exclusion criteria

During the experiment, any mouse that died as a direct result of the ICV injection procedure was excluded from the study.

### Immunofluorescence staining

The harvested mouse brains were fixed in 4% PFA solution at 4 °C overnight. Subsequently, the fixed brain tissues were dehydrated by successive dipping in 20% and 30% sucrose solutions at 4 °C until the brains sank. Then, the brain tissues were embedded in OCT compound before being cut into 20-μm-thick sections on a cryostat (Leica Microsystems, Wetzlar, Germany) at −19 °C. The cryosections were permeabilized (1X PBS/0.2% Triton X-100) for 30 min and incubated with blocking solution (1× PBS/0.1% Triton X-100/10% normal horse serum) for 1 h at room temperature. After blocking, the cryosections were incubated with a rabbit anti-CD34 antibody (1:500, ab81289, Abcam, Cambridge, MA, USA) overnight at 4 °C. After primary antibody incubation, the cryosections were rinsed three times with PBS and incubated with a rabbit 647-conjugated anti-rabbit antibody (1:1000; Cell Signaling Technology, Beverly, MA, USA) for 1 h at room temperature in the dark. Then, the cryosections were rinsed three times, and 4′,6-diamidino-2-phenylindole (DAPI) reagent was used to stain the nucleus (1 μg/mL) for 10 min. After DAPI staining, the cells were rinsed three times, and coverslips were mounted on glass slides with antifade reagent. Then, images were taken using the laser confocal microscope (Carl Zeiss; Jena, Germany). Results were expressed as the mean ± SEM of three independent experiments.

### Immunocytochemical staining

For immunocytochemical staining of ZO-1, the cells cultured on coverslips were transfected with the miR-103b mimic or its negative control for 72 h. Then the cells were subsequently rinsed three times with PBS and fixed with 4% PFA for 15 min at room temperature. After fixation, the cells were rinsed three times with PBS and then blocked in blocking buffer (1× PBS/5% horse serum/0.1% Triton X-100) for 1 h. After blocking, the cells were incubated with a rabbit anti-ZO-1 antibody (1:500, Cell Signaling Technology, Beverly, MA, USA) overnight at 4 °C. After primary antibody incubation, the cells were rinsed three times with PBS and incubated with a rabbit 647-conjugated anti-rabbit antibody (1:1000; Cell Signaling Technology, Beverly, MA, USA) for 1 h at room temperature in the dark. Then, the cells were rinsed three times, and DAPI reagent was used to stain the nucleus (1 μg/mL) for 10 min. After DAPI staining, the cells were rinsed three times, and coverslips were mounted on glass slides with antifade reagent. Then, cell images were taken using the laser confocal microscope (Carl Zeiss; Jena, Germany). Results were expressed as the mean ± SEM of three independent experiments.

### Live-cell imaging assay of fluorescently labeled exosome uptake

For the detection of real-time uptake of exosomes, the cells were cultured in L-15 medium supplemented with 10% exosome-depleted FBS. The isolated exosomes were labeled with PKH67 according to the manufacturer's protocol (Sigma-Aldrich, USA). One microgram of PKH67-labeled exosomes was added to cells and incubated at 37 °C for 2 h. The real-time uptake of PKH67-labeled exosomes was captured every 5 min for 30 min using a DeltaVision Elite Cell imaging system (Applied Precision Inc., GE Healthcare Company, USA).

### Cell viability assay

Cell viability was measured using the CCK-8 reagent (Dojindo Laboratories, Japan) following the manufacturer's instructions.

### Apoptosis detection

Apoptotic cells were examined using Annexin V, FITC Apoptosis Detection Kit (Dojindo Laboratories, Japan) following the manufacturer's instructions. Then, cell images were taken using the Operetta High-content Imaging System (HCIS) (PerkinElmer, USA), and the mean fluorescence intensity was determined using Harmony Software (PerkinElmer, USA).

### Intracellular ROS and mitochondria detection

Intracellular ROS was detected using DCFH-DA reagent (Dojindo Laboratories, Japan) according to the manufacturer's instructions. Mitochondria were stained with MitoBright LT Deep Red (Dojindo Laboratories, Japan) according to the manufacturer's instructions. Then, cell images were taken using the Operetta HCIS (PerkinElmer, USA), and the mean fluorescence intensity was determined using Harmony Software (PerkinElmer, USA).

### MicroRNA sequencing and mRNA sequencing

The HUVECs and U251 cells transfected with miR-103b mimic or its negative control miRNA underwent mRNA sequencing. Exosomes derived from MDA-MB-231 cells and MDA-MB-231Brm cells underwent miRNA sequencing. All the samples were sent to Sinotech Genomics Corporation (Shanghai, China) for RNA extraction and cDNA library construction, and Illumina NovaSeq 6000 was used for high-throughput RNA sequencing. Three samples from each group were sequenced. Clean reads were obtained by removing adapter sequences, ribosomal RNA reads, reads with a length < 25 nt, empty reads, reads containing unknown sequences “N” with a rate of more than 10%, and low-quality reads containing more than 50% bases with quality score Q values ≤ 5 from the raw reads. Clean reads were mapped to the Homo sapiens GRCh38.100 genome sequence using HISAT2 (V2.0.4, The Center for Computational Biology at Johns Hopkins University, Baltimore, MD). To compare the gene expression levels, the expression of the reads was converted into FPKM (fragments per kilobase of exon model per million mapped reads) for gene expression standardization using StringTie (version 1.3.0, The Center for Computational Biology at Johns Hopkins University, Baltimore, MD) and then normalized with the TMM (trimmed mean of M values) method. *p*-values were obtained using edgeR to analyze the differential gene expression between samples. Then, multiple hypothesis test correction was performed to determine the threshold of the *p*-value by controlling the false discovery rate. The corrected *p*-value is the Q value. Fold change (FC) in differential expression was calculated according to the FPKM value. The differential gene screening conditions were determined by a Q value < 0.05 and an absolute value of log2 (FC) in expression greater than or equal to 1. The sequencing data are available in the GEO repository (accession number GSE320274).

### RNA extraction and quantitative PCR analysis (qPCR)

Total RNA extraction was performed using TRIzol reagent (Invitrogen, Carlsbad, CA, USA) according to the manufacturer's instructions. cDNA was reverse transcribed from total RNA using the PrimeScript™ RT reagent Kit with gDNA Eraser (Takara, Japan) according to the manufacturer's instructions. Then, the TB Green qPCR kit (Takara, Japan) and an iCycler iQ thermal cycler (BioRad) were used for analyzing the mRNA expressions (HPRT1 as an internal control). Change in mRNA level was quantified using the 2^−^^ΔΔCT^ method. The primers were listed in Table 1.

**Table 1.** Primer of qPCR.

Gene	Forward	Reverses
HPRT1	5′-CCCTGGCGTCGTGATTAGTG-3′	5′-TCGAGCAAGACGTTCAGTCC-3′
TJP1	5′-TCGGAAAAGTGCCAGGAAGT-3′	5′-TTGTAGCACCATCCGCCTTT-3′
ACTB	5′-AGGATTCCTATGTGGGCGAC-3′	5′-GGTCTCAAACATGATCTGGGT-3′
PLK1	5′-CCATCACCTGCCTGACCATT-3′	5′-GGGGGTTCTCCAAGCCTTTA-3′
ZNF768	5′-GGTACCTCAGAGGCAACAT-3′	5′-GGGTTCAAGCCCAAATGGTA-3′
ACTG1	5′-CCTTCCTGGGTATGGAATCT-3′	5′-CTTCTGCATCCTGTCGGCAA-3′
SDHC	5′-GCTGTTGCTGAGACACGTT-3′	5′-GACATCGCCATGGGAAGAGA-3′
SLC2A1	5′-TGGCATCAACGCTGTCTTCT-3′	5′-AGAAGACAGCGTTGATGCCA-3′
GADD45B	5′-ATCGCCCTGCAAATCCACTT-3′	5′-GTCCGTGTGAGGGTTCGTG-3′
TNFSF10	5′-TGAAGCAGATGCAGGACAAG-3′	5′-TCTTTCTAACGAGCTGACGG-3′

### Bioinformatic analysis

Enrichment of biological processes was analyzed using the GO database (http://www.geneontology.org/). The enrichment of the signaling pathway was analyzed using the KEGG database (https://www.kegg.jp/). Reactome pathway and Wikipathway (PPI) network were obtained by STRING7 (https://string-db.org) (confidence score > 0.4) and visualized by Cytoscape 3.5.0.

### Gene targets analysis

Bioinformatic target analysis was done using four publicly available algorithms, including miRBase V22.1 (https://www.mirbase.org/), TargetScan8.0 (https://www.targetscan.org/vert_80/), miRDB (http://mirdb.org/), and miRWalk (http://mirwalk.umm.uni-heidelberg.de/).

### Statistical analysis

All the experiments were performed with three independent biological replicates unless otherwise stated. For *in vitro* experiments, each independent biological replicate consisted of cells from a separate passage or a separately thawed vial. For *in vivo* experiments, each biological replicate represented an individual mouse. In the miR-negative-3′chol injection group, one mouse died after ICV injection and was excluded from the analysis. In the miR-103b-3′chol injection group, two mice died after ICV injection and were excluded from the analysis. Randomization was performed using a random number generator. The experimenter who conducted the outcome assessments was blinded to group allocation. The exact number of replicates (*n*) and the number of independent experiments are specified in each figure legend. Technical replicates were performed as indicated but were averaged per biological replicate for statistical analysis. For in vivo experiments, before statistical analysis, we assessed whether the data met the assumptions of the parametric tests. Normality was assessed using the Shapiro‒Wilk test. The data from all the experimental groups were found to follow a normal distribution (*p* > 0.05 for all the groups: control group: *p* = 0.094; miR-103b group: *p* = 0.247). Therefore, parametric tests were used without any transformation. The results are represented as mean ± SEM of independent experiments. Two-sample unpaired *t*-tests or one-way analysis of variance (ANOVA) followed by Bonferroni post hoc test for multiple comparisons were performed for statistical analysis using GraphPad Prism 5. Significant differences were assigned to *p*-values < 0.05,<0.01 and <0.001, denoted by *, **, and ***, respectively. For GO enrichment analysis, Fisher's exact method was used to calculate the significance level of each gene.

## Results

### miR-103b was significantly increased in the exosomes derived from MDA-MB-231Brm cells

To obtain exosomal miRNA, exosomes were isolated from the conditioned culture medium of MDA-MB-231 cells and MDA-MB-231Brm cells and visually confirmed by TEM. The results showed that the isolated extracellular particles were approximately 100 nm in size and exhibited the typical exosomal cup-shaped morphology (Figure 1A). Furthermore, the uptake of exosomes derived from MDA-MB-231Brm cells by HUVECs, a widely used surrogate for BMECs in an *in vitro* BBB model,^23^ was visually confirmed by live‐cell microscopy. The results showed that PKH67-labeled exosomes derived from MDA-MB-231Brm cells can be taken up by HUVECs (Figure 1B and Supplement S1). This result suggests that the uptake of miRNA cargo in exosomes derived from MDA-MB-231Brm cells by constituent cells of the BBB might modulate the formation of PMN and mediate TNBC-BM. Then, to identify the key exosomal miRNA regulating BBB disruption in the pathology of TNBC-BM, the secretome of exosomal miRNA was analyzed using miRNA-seq. The results revealed 150 differentially secreted miRNAs (DSMis) (|log2FC| ≥ 0.5; FDR value *<* 0.05), of which 14 were up-secreted (known miRNA: 6, novel miRNA: 8) and 136 were down-secreted (known miRNA: 111, novel miRNA: 25) in the exosomes derived from MDA-MB-231Brm cells compared to those derived from MDA-MB-231 cells (Figure 1C and Supplement S2). Notably, among 14 up-secreted DEMis, miR-103b was the only DSMis that was not only the most abundant miRNA (TPM ≥ 100) but also the most significantly up-secreted miRNA (|log2FC| ≥ 1) (Figure 1D). Furthermore, qPCR analysis also confirmed the obvious increase in miR-103b expression in the exosomes derived from MDA-MB-231Brm cells Compared to its **parental** counterpart (Figure 1E).

**Figure 1.**
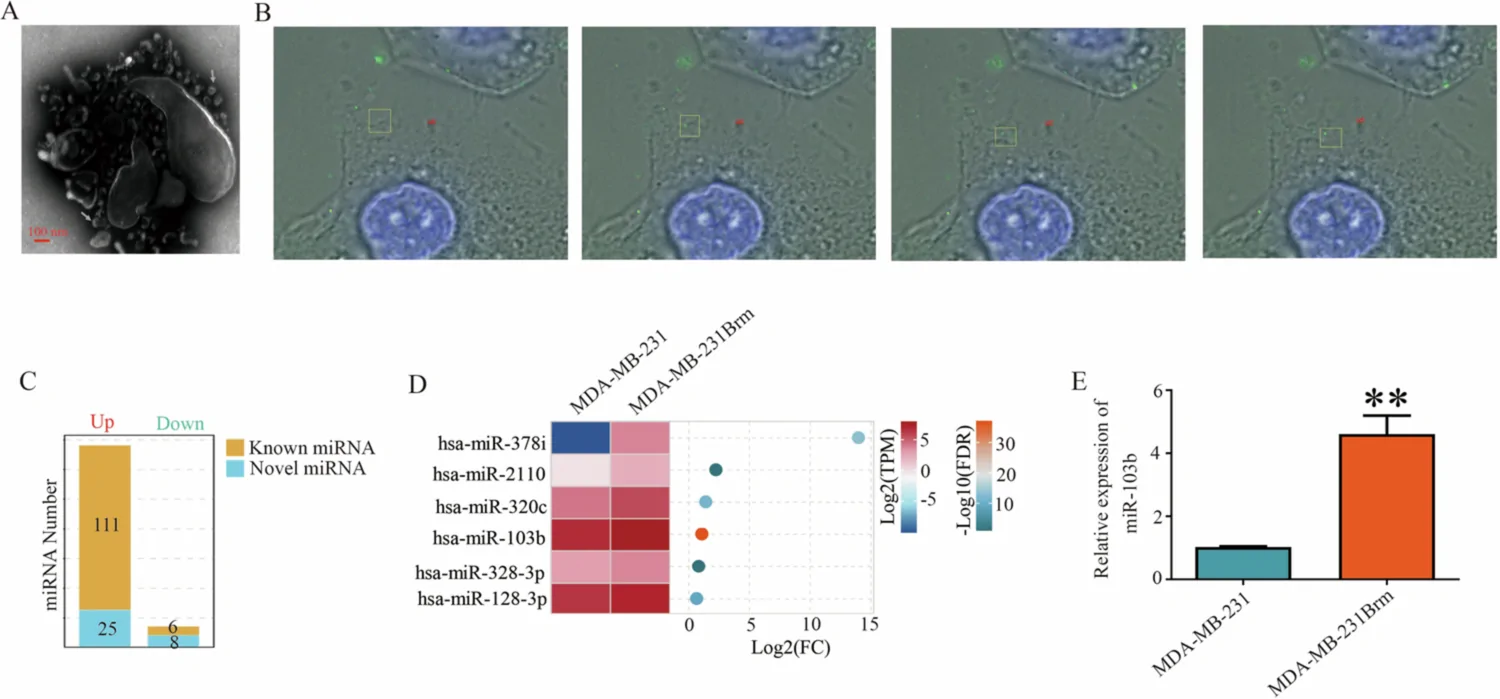
miR-103b was significantly elevated in the exosomes derived from MDA-MB-231Brm cells. (A) Representative electron microscope images of isolated exosomes. Scale bar = 100 nm. (B) Representative live-cell microscopy images of the uptake of exosomes by HUVECs. (C) Statistical data of the number of differentially secreted miRNAs in exosomes derived from MDA-MB-231 versus MDA-MB-231Brm cells. Up: up-secreted miRNAs, Down: down-secreted miRNAs. (D) Heatmap showing the expression value of six up-secreted known miRNAs. (E) qPCR statistical data of the relative expression of miR-103b in exosomes derived from MDA-MB-231 versus MDA-MB-231Brm cells. Data are expressed as mean ± SEM from three independent experiments; **p < 0.01.

### miR-103b compromised the tight junction of HUVECs and triggered the increased permeability of the BBB in mice

To determine whether increased miR-103b in the exosomes derived from MDA-MB-231Brm cells modulates the formation of brain PMN, we first explored the impact of miR-103b on the disruption of the tight junction of the BBB, an important pathological marker in TNBC-BM PMN, using HUVEC cells. The results demonstrated that ZO-1-labeled tight junctions were significantly reduced in HUVECs transfected with miR-103b-mimic compared to those in HUVECs transfected with the negative control miRNA (miR-NC) (Figure 2A and B). Furthermore, the effect of miR-103b on the permeability of the BBB was examined *in vivo* by injecting cholesterol-modified miR-103b into the lateral ventricle of C57BL/6 mice. First, we tracked the distribution of cholesterol-modified miRNA in the brain of C57BL/6 mice after lateral ventricle injection using 5′-Cy3-miR-negative-3′ cholesterol. It was observed that the injected miRNA entered the brain tissue and was mainly distributed alongside the lateral ventricles 7 d after injection (Figure 2C). Then, the permeability of the BBB was examined using FITC-Dextran. As expected, FITC-Dextran obviously increased in the brain of the mice injected with miR-103b-mimic (mean ± SEM:21.53 ± 1.01) compared to that in the brain of the mice injected with miR-NC (mean ± SEM:10.76 ± 0.69) (Figure 2D and E). These results provide the possibility that increased miR-103b in exosomes derived from TNBC-BM cells compromises the integrity of the BBB and increases its permeability.

**Figure 2.**
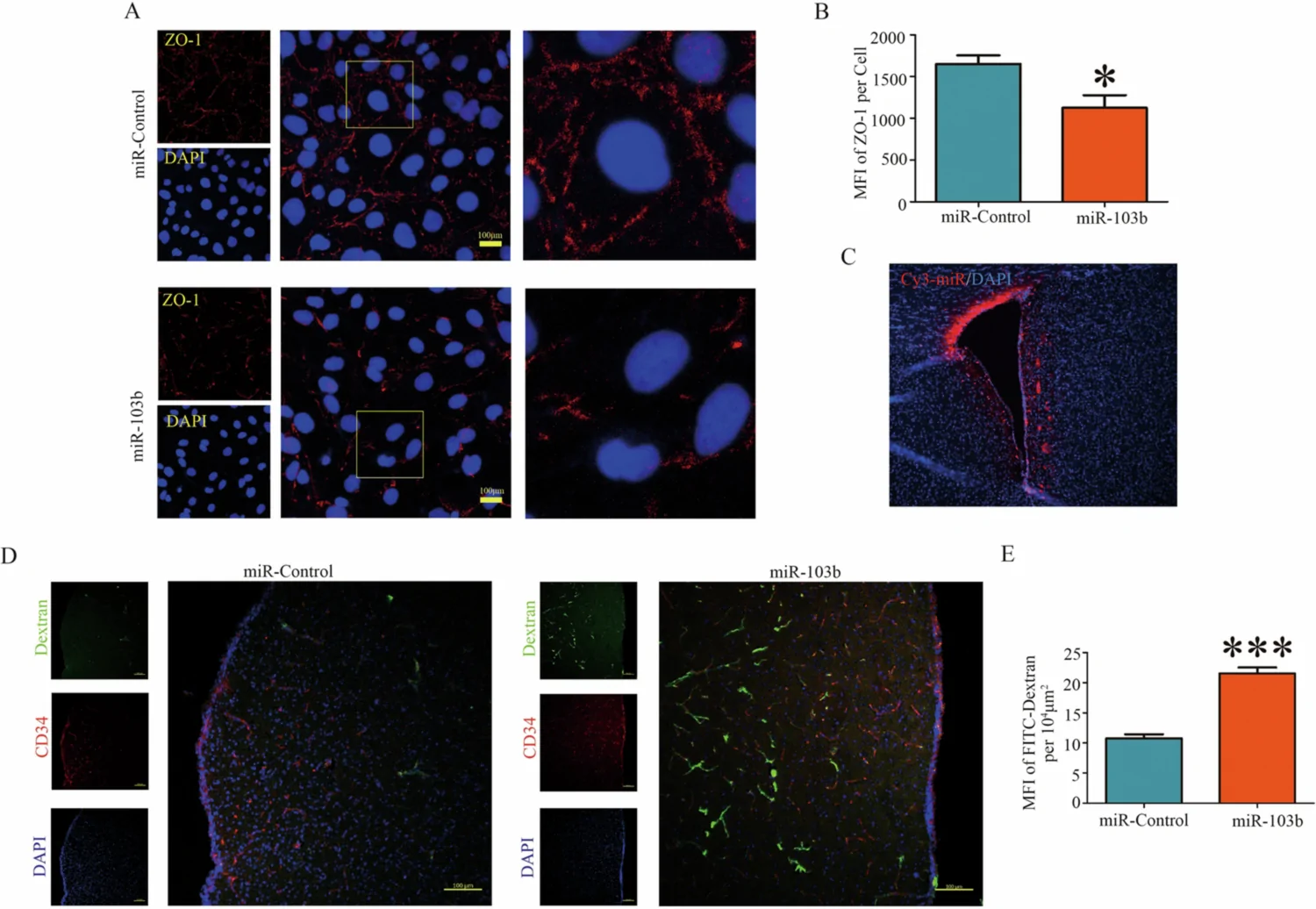
miR-103b compromised the tight junction of HUVECs and increased permeability of the BBB in mice. (A) Representative immunocytochemical staining images of ZO-1 in HUVECs transfected with miR-103b mimic (miR-103b) or negative control miRNA (miR-Control). The nucleus was stained with DAPI; scale bar = 100 μm. (B) Statistical data of (A) showing the mean fluorescence intensity (MFI) of ZO-1. (C) Representative staining image showing the distribution of injected 5′-Cy3-labeled miRNA in the brain of the mice. The nucleus was stained with DAPI. (D) Representative immunofluorescence staining image showing the extravasation of FITC-labeled dextran in the brain of mice injected with a cholesterol-conjugated miR-103b mimic (miR-103b) or cholesterol-conjugated negative control miRNA (miR-Control). Vessels were stained with CD34, and the nucleus was stained with DAPI; scale bar = 100 μm. miR-control group:n = 7, miR-103b group: *n* = 6. (E) Statistical data of (D) showing relative FITC mean fluorescence intensity (MFI) within the same region of interest (ROI) of $10^{4}\;{\mathrm{\mu m}}^{2}$ . The data are expressed as mean ± SEM from three independent experiments; *p < 0.05, ***p < 0.001.

### miR-103b inhibited the cell viability of the HUVECs and the human astrocyte cell line U251 cells

The increased permeability of the BBB implies compromised structural integrity. BMECs and astrocytes, the two primary cellular constituents of the BBB, play a critical role in preserving the integrity and functionality of the BBB. Their intricate partnership is essential for protecting the brain and ensuring a stable internal environment.^19^ Based on the above results, we wondered about the impact of increased miR-103b in the exosomes derived from MDA-MB-231Brm cells on the cell components of the BBB.

Given the above exosomal uptake result confirmed by live-cell imaging, to more clearly delineate the specific effects of miR-103b and to exclude potential confounding influences from other exosomal cargos—such as proteins, lipids, and other non-coding RNAs that may independently modulate recipient cell phenotypes—we chose to directly transfect miR-103b mimics instead of exosomes derived from miR-103b-overexpressing parental cells into HUVECs and U251 cells, two cell lines commonly employed as surrogate cell lines for brain microvascular endothelial cells and astrocytes.

First, the CCK-8 assay revealed that miR-103b significantly inhibited the viability of HUVECs and U251 cells (Figure 3A and B). Meanwhile, the apoptosis assay revealed that miR-103b triggered an obvious increase in both early (annexin V+/PI−) and late (annexin V+/PI+) apoptosis in HUVECs and U251 cells (Figure 3C–F). These results hint that increased miR-103b in exosomes derived from TNBC-BM cells could compromise the structural integrity of the BBB by inducing apoptosis in BMECs and astrocytes.

**Figure 3.**
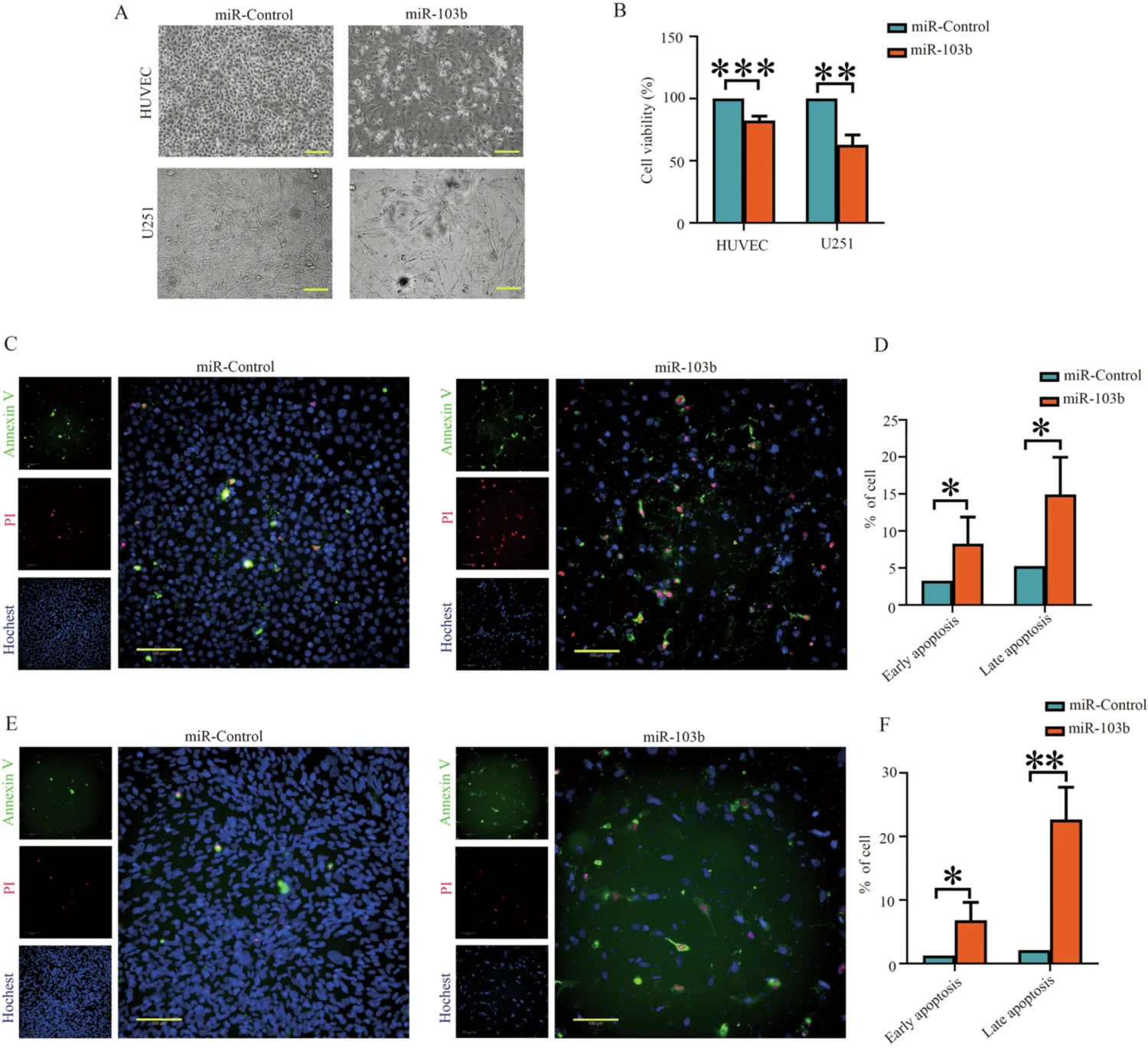
miR-103b induced apoptosis in HUVECs and U251 cells. (A) Representative light microscopy images of the cell viability of HUVECs and U251 cells transfected with miR-103b mimic (miR-103b) or negative control miRNA (miR-Control). Scale bar = 100 μm. (B) Cell viability of (A) was quantified using the CCK-8 assay. (C) Representative HCIS images of the Annexin V/PI staining showing the apoptotic cells in HUVECs transfected with miR-103b mimic (miR-103b) or negative control miRNA (miR-Control). The nucleus was stained with Hoechst 33342 (Hoechst); scale bar = 100 μm. (D) Statistical data of (C) showing the percentage of early apoptotic cells and late apoptotic cells, respectively. (E) Representative HCIS images of the Annexin V/PI staining showing the apoptotic cells in U251 cells transfected with miR-103b mimic (miR-103b) or negative control miRNA (miR-Control). The nucleus was stained with Hoechst 33342 (Hoechst); scale bar = 100 μm. (F) Statistical data of (E) showing the percentage of early apoptotic cells and late apoptotic cells, respectively. Data are expressed as mean ± SEM from three independent experiments; *p < 0.05, **p < 0.01, ***p < 0.001.

### miR-103b elevated the intracellular ROS levels in both HUVECs and U251 cells

Given the observed pro-apoptotic role of miR-103b, we next investigated the effect of miR-103b on the intracellular oxidative stress, a major mediator of apoptosis, in HUVECs and U251 cells. ROS-sensitive DCFH-DA staining revealed a significant increase in the intracellular ROS levels in both miR-103b-mimic-transfected HUVECs and U251 cells compared to those in their miR-NC-transfected counterparts (Figure 4A–D). Dysfunctional mitochondria are suggested as a major source of intracellular ROS.^24^ ^,^ ^25^ We then probed mitochondrial alterations using MitoBright Deep Red, a fluorescent probe for the selective staining of mitochondria. The results clearly revealed a markedly increased fluorescence intensity per cell in both miR-103b-mimic-transfected cell lines (Figure 4E–H). Thus, these results indicated that increased miR-103b in the exosomes derived from TNBC-BM cells may cause mitochondrial dysfunction and ROS accumulation in BMECs and astrocytes.

**Figure 4.**
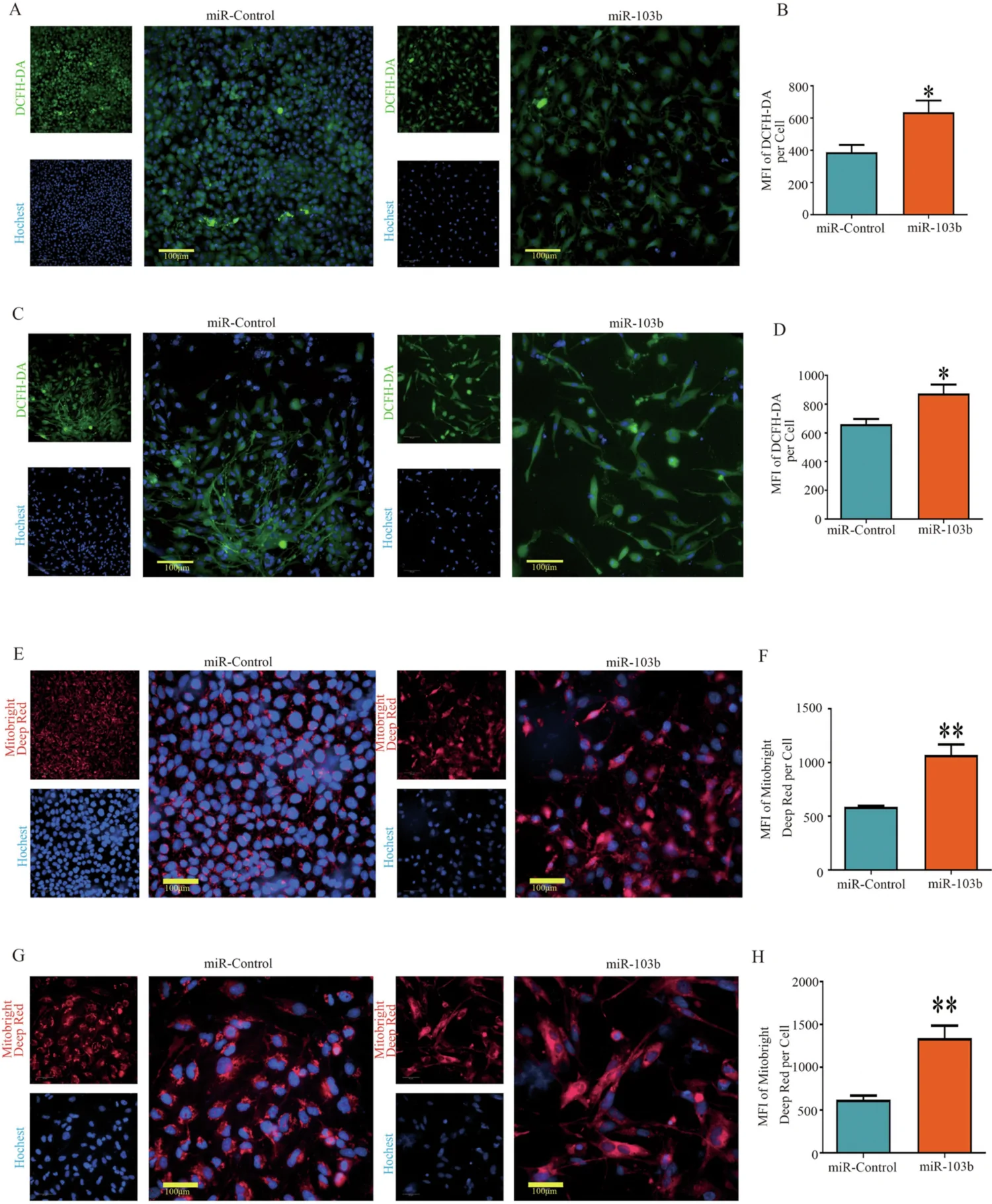
miR-103b increased the intracellular ROS levels in both HUVECs and U251 cells. (A) Representative HCIS images of the DCFH-DA staining in HUVECs transfected with miR-103b mimic (miR-103b) or negative control miRNA (miR-Control). The nucleus was stained with Hoechst 33342 (Hoechst); scale bar = 100 μm. (B) Statistical data of (A) showing MFI of DCFH-DA per cell. (C) Representative HCIS images of the DCFH-DA staining in U251 cells transfected with miR-103b mimic (miR-103b) or negative control miRNA (miR-Control). The nucleus was stained with Hoechst 33342 (Hoechst); scale bar = 100 μm. (D) Statistical data of (C) showing MFI of DCFH-DA per cell. (E) Representative HCIS images of the Mitobright Deep Red staining in HUVECs transfected with miR-103b mimic (miR-103b) or negative control miRNA (miR-Control). The nucleus was stained with Hoechst 33342 (Hoechst); scale bar = 100 μm. (F) Statistical data of (E) showing MFI of Mitobright Deep Red per cell. (G) Representative HCIS images of the Mitobright Deep Red staining in U251 cells transfected with miR-103b mimic (miR-103b) or negative control miRNA (miR-Control). The nucleus was stained with Hoechst 33342 (Hoechst); scale bar = 100 μm. (H) Statistical data of (G) showing MFI of Mitobright Deep Red per cell. The data are expressed as mean ± SEM from three independent experiments; *p < 0.05, **p < 0.01.

### Identification of differentially expressed genes (DEGs) regulated by miR-103b in the HUVECs and U251cells

Given the above results, to gain a comprehensive underlying molecular understanding, we further investigated the miR-103b-induced transcript changes in HUVECs and U251 cells using high-throughput RNA-sequencing. The results revealed 2569 DEGs (Q value < 0.05, |log2FC| ≥ 0.5, average FPKM ≥ 1), among which 1415 were down-regulated, and 1154 were up-regulated in the miR-103b-mimic-transfected HUVECs compared to the miR-NC-transfected HUVECs (Figure 5A and Supplement S3). Among the downregulated genes, 19 were identified as the most significantly decreased, with log2FC ≤ −2.5, including IGFBP3, TNFSF14, SUN2, TMEM88, KIFC1, GAL3ST1, TEKTIP1, H3C14, S100A2, CDCA3, AURKA, MND1, TMSB15A, CDCA5, ORC1, KIF20A, TYMS, BEST1, and CDC25C, among which 9 genes, including SUN2, ORC1, MND1, KIFC1, KIF20A, CDCA5, CDC25C, CDCA3, and AURKA, were cell cycle-associated genes. (Figure 5B and Supplement S3). Among the upregulated genes, 20 genes were identified as the most significantly increased, with log2FC ≥ 2.5, including IFI44L, IFI44, IFIT1, OASL, IFI6, IFIT3, BST2, IFIT2, TRIM34, AKAP5, GBP4, MMP24, CPLX1, C1S, SNED1, RHEX, ISG15, OVGP1, DDX60, and NT5E, among which 9 genes, including IFIT1, OASL, IFI6, IFIT3, IFIT2, TRIM34, GBP4, BST2, and ISG15, were interferon signaling genes (Figure 5C and Supplement S3). In the miR-103b-mimic-transfected U251 cells, high-throughput RNA-sequencing revealed 3298 DEGs (Q value < 0.05, |log2FC| ≥ 0.5, average FPKM ≥ 1), among which 1784 were downregulated and 1514 were upregulated compared to those in the miR-NC-transfected U251 cells (Figure 5D and Supplement S4). Among the downregulated genes, 17 genes were identified as the most significantly decreased, with log2FC ≤ −2.5, including HAS2, RGS8, HBB, HBA2, HBA1, EGR1, RTL3, SLC38A3, FGFBP2, DKK1, ZNF804A, MT-ND6, CEMIP, OLFML2A, GAS1, MT-ATP6, and INKA2. (Figure 5E and Supplement S4). Among the upregulated genes, 25 genes were identified as the most significantly increased, with log2FC ≥ 2.5, including XAF1, HLA-F, CXCL11, DHRS2, PSMB9, CXCL10, CDRT4, H2BC18, H2BC4, APOL1, PLAAT4, IFI6, NT5E, PTX3, H2AC18, H2BC11, RSAD2, IGFBP6, H4C8, OASL, IL4I1, H2BC9, GCH1, C3, and H2AC19 (Figure 5F and Supplement S4).

**Figure 5.**
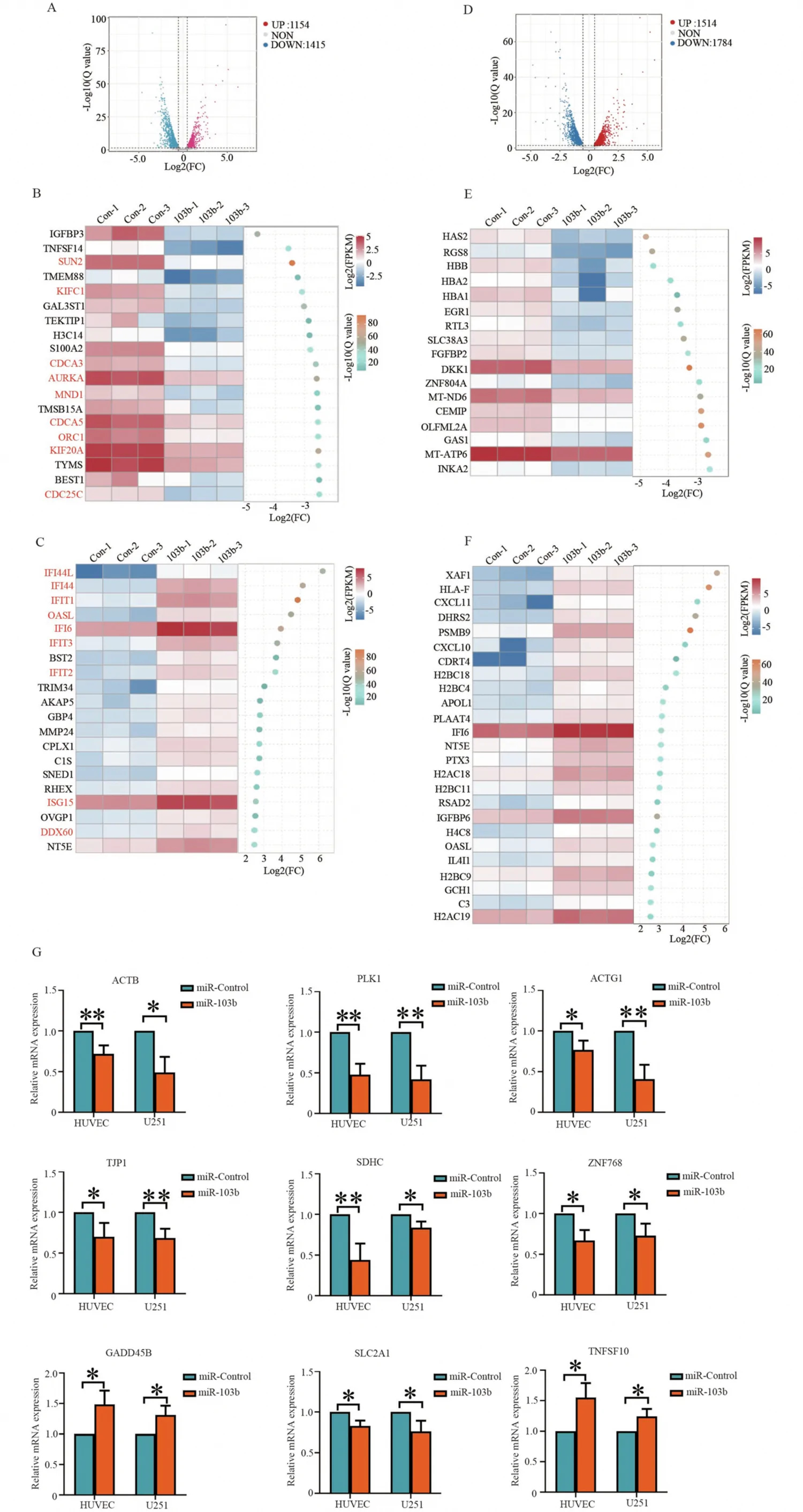
Profiling of miR-103b-regulated DEGs in HUVECs and U251 cells. (A) Volcano plots showing the distribution of 2569 DEGs (|log2FC| ≥ 0.5; average FPKM ≥ 1, *Q*-value < 0.05) in HUVECs regulated by miR-103b. Up: up-regulated genes, Down: down-regulated genes. (B) Heat map showing the expression of the top nineteen down-regulated genes with log2FC ≤ −2.5 in HUVECs. Nine cell-cycle-associated genes were marked in red. (C) Heat map showing the expression of the top twenty up-regulated genes with log2FC ≥ 2.5. Nine interferon signaling-associated genes were marked in red. (D) Volcano plots showing the distribution of 3298 DEGs (|log2FC| ≥ 0.5; average FPKM ≥ 1, *Q*-value < 0.05) in U251 cells regulated by miR-103b. Up: up-regulated genes, Down: down-regulated genes. (E) Heat map showing the expression of the top seventeen down-regulated genes with log2FC ≤ −2.5 in U251 cells. (F) Heat map showing the expression of the top twenty-five up-regulated genes with log2FC ≥ 2.5 in U251 cells. (G) The mRNA expression levels of selected genes were determined by a qPCR experiment. HPRT1 as an internal control. The data are expressed as mean ± SEM from three independent experiments; *P < 0.05, **p < 0.01.

To validate the RNA-seq findings, qPCR was conducted to examine the expression of TJP1, ACTB, PLK1, ACTG1, ZNF768, SDHC, SLC2A1, GADD45B, and TNFSF10 in HUVECs and U251 cells transfected with either miR-103b-mimic or miR-NC. As shown in Figure 5G, the qPCR results confirmed the changes in transcript levels observed in the RNA-seq analysis, which were validated the reliability of the sequencing data (Figure 5G).

### GO analysis of DEGs regulated by miR-103b in HUVECs and U251 cells

To identify the functional classifications of DEGs in HUVECs and U251 cells, functional enrichment analysis was performed using the GO database. The results showed that DEGs in HUVECs were enriched mainly in the meiotic cell cycle-associated biological process (BP), DNA damage and repair-associated BP, regulation of cell-cell junction, regulation of extracellular matrix-associated BP, apoptosis-associated BP, and cell proliferation-associated BP, and so on (Figure 6A and Supplement S5). Being different from DEGs in HUVECs, DEGs in U251 cells were enriched mainly in glucose metabolic process-associated BP, oxidative phosphorylation-associated BP, NADPH metabolic process-associated BP, apoptosis-associated BP, establishment of the endothelial intestinal barrier, protein localization to cell-cell junction, cell cycle-associated BP, and so on (Figure 6B and Supplement S6). These observations indicate that increased miR-103b in TNBC-BM exosomes could trigger different cell biological processes in BBB BMECs and astrocytes.

**Figure 6.**
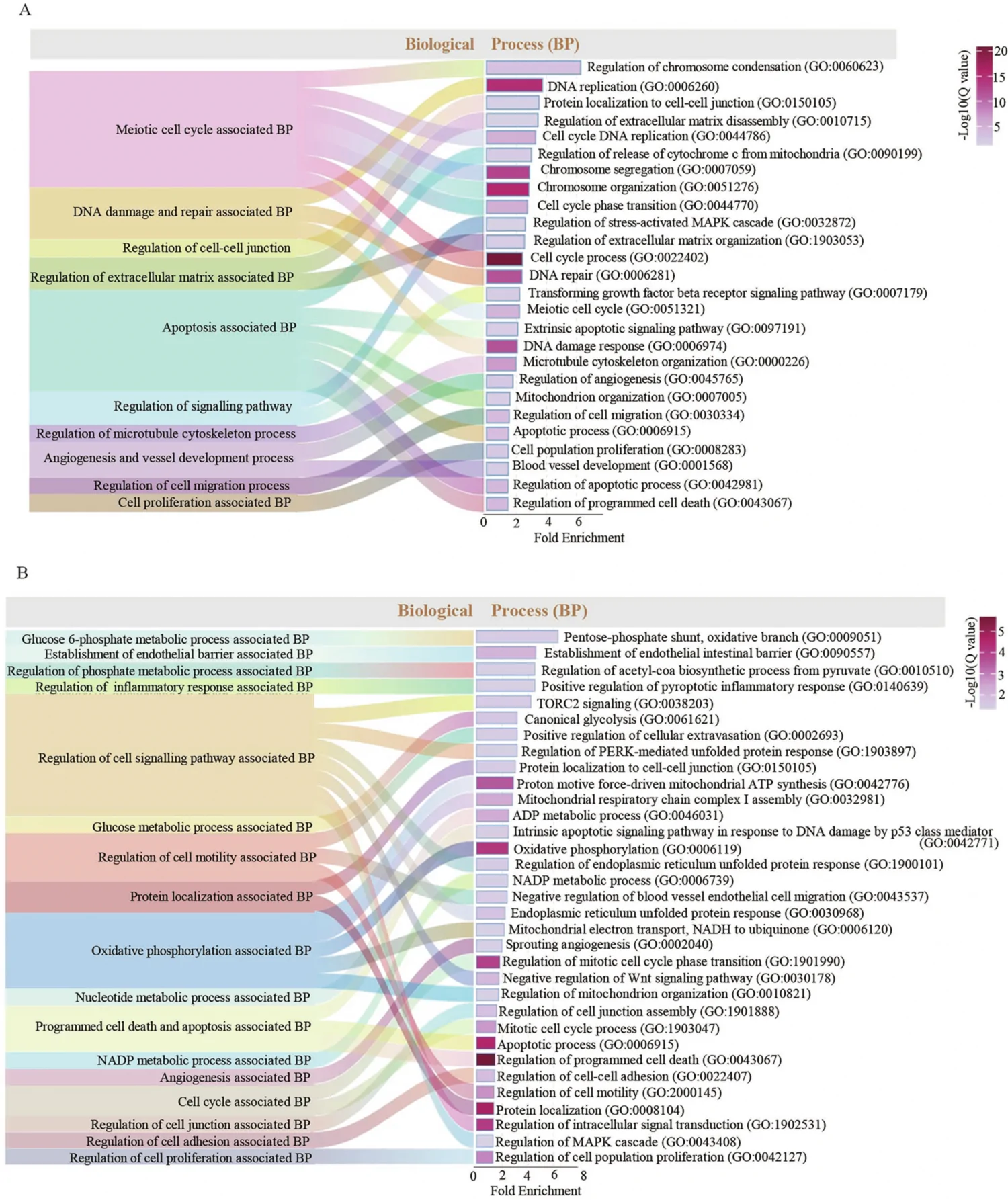
GO analysis of miR-103b-regulated DEGs in HUVECs and U251 cells. (A) Sankey plot showing the biological process enrichment of miR-103b-regulated DEGs in HUVECs. (B) Sankey plot showing the biological process enrichment of miR-103b-regulated DEGs in U251 cells.

### KEGG analysis of DEGs regulated by miR-103b in HUVECs and U251 cells

KEGG pathway analysis showed that both DEGs in HUVECs and DEGs in U251 cells were involved in multiple KEGG pathway categories. In the cellular processes category, both groups of DEGs were significantly enriched in pathways such as cell apoptosis, the cell cycle, cellular senescence, the p53 signaling pathway, and tight junction (Figure 7A and Supplements S7, S8). In the environmental information processing category, DEGs from both HUVECs and U251 cells were notably enriched in the MAPK, FoxO, and Hippo signaling pathways. Additionally, DEGs in HUVECs were enriched in the TNF, TGF-β, and ErbB signaling pathways, as well as ECM-receptor interaction. Conversely, DEGs in U251 cells were enriched in the Notch, AMPK, and HIF-1 signaling pathways (Figure 7B and Supplement S7, S8). In the genetic information processing category, DEGs from both cell types were significantly enriched in pathways related to DNA replication, mismatch repair, ubiquitin-mediated proteolysis, proteasome, homologous recombination, base excision repair, nucleotide excision repair, and the Fanconi anemia pathway (Figure 7C and Supplement S7, S8). However, in the metabolism category, the pathways enriched by DEGs in HUVECs and U251 cells were quite different. DEGs from HUVECs were enriched mainly in biosynthetic pathways, including pyrimidine metabolism, vitamin B6 metabolism, steroid biosynthesis, and terpenoid backbone biosynthesis. In contrast, DEGs from U251 cells were enriched primarily in energy metabolism pathways such as oxidative phosphorylation, fatty acid metabolism, the TCA cycle, glycolysis/gluconeogenesis, and the biosynthesis of unsaturated fatty acids (Figure 7D and Supplements S7, S8). The key node molecules within the KEGG network for HUVECs included TRAF2, TNFRSF1A, PCNA, NFKBIA, MAPK8, MAPK3, JUN, HRAS, GADD45B, CHUK, CASP8, and BIRC2 (Figure 7E; Supplementary S7, S8). For U251 cells, important node molecules included PFKP, KRAS, PFKL, CCND1, AKT1, PIK3R2, PIK3CD, MAPK1, MAPK3, ACTB, and ACTG1 (Figure 7F and Supplementary S7, S8).

**Figure 7.**
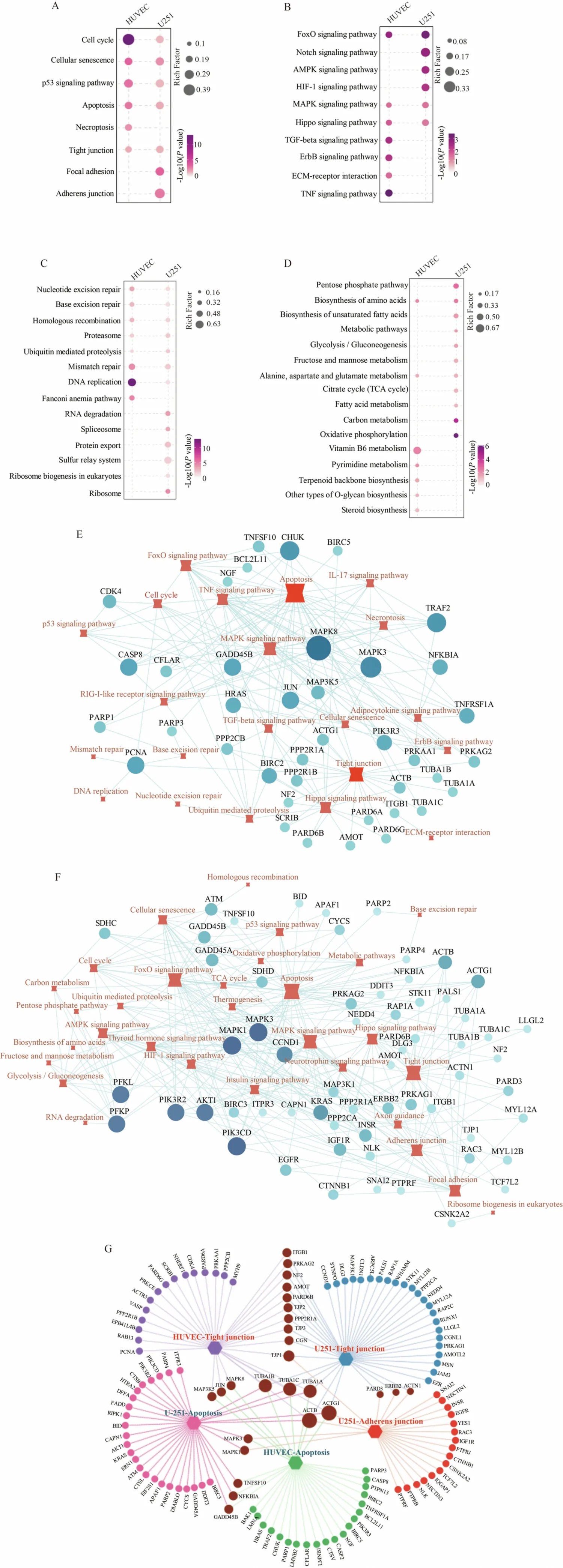
KEGG analysis of the miR-103b-regulated DEGs in the HUVECs and U251 cells. (A) Dot plot showing the enriched signaling pathway of miR-103b-regulated DEGs in the cellular processes class in HUVECs and U251 cells (U251). (B) Dot plot showing the enriched signaling pathway of miR-103b-regulated DEGs in the environmental information processing class in HUVECs and U251 cells (U251). (C) Dot plot showing the enriched signaling pathway of miR-103b-regulated DEGs in the genetic information processing class in HUVECs and U251 cells (U251). (D) Dot plot showing the enriched signaling pathway of miR-103b-regulated DEGs in the metabolism class in HUVECs and U251 cells (U251). (E) Network image showing the pathway interaction and its main molecules in HUVECs. (F) Network image showing the pathway interaction and its main molecules in U251 cells. (G) Network Venn diagram showing the overlap of genes involved in the apoptosis and tight junction pathways in HUVECs and U251 cells.

Given the above pro-apoptotic and tight junction-regulating effects of miR-103b, we interrogated the DEGs associated with these two pathways. Apoptosis pathway analysis identified 32 DEGs in HUVECs and 34 in U251 cells, with 9 genes shared between them: GADD45B, NFKBIA, TNFSF10, TUBA1C, TUBA1B, TUBA1A, ACTG1, ACTB, and MAPK3. Tight junction pathway analysis identified 33 DEGs in HUVECs and 41 in U251 cells, with 15 genes shared between them: CGN, TJP3, PPP2R1A, TJP2, PARD6B, AMOT, NF2, PRKAG2, ITGB1, TJP1, TUBA1C, TUBA1B, TUBA1A, ACTG1, and ACTB (Figure 7G and Supplement S7, S8). The results indicate that cytoskeletal genes, including TUBA1C, TUBA1B, TUBA1A, ACTG1, and ACTB, could be important target molecules in miR-103b-mediated apoptosis and the tight junction pathway in BBB BMECs and astrocytes.

### Inflammatory cytokines analysis of DEGs regulated by miR-103b in HUVECs and U251 cells

Pro-inflammatory cytokines/chemokines are key mediators in the formation of brain PMN.^26^ ^,^ ^27^ Given the above KEGG enrichment containing multiple inflammation-associated pathways, such as TNF, TGF-β, and MAPK signaling pathways, we further investigated the network of miR-103b-induced pro-inflammatory cytokines/chemokines in HUVECs and U251 cells using the GeneCards database. The results demonstrated that miR-103b obviously upregulated 186 and 196 pro-inflammatory cytokine/chemokine-related genes in HUVECs and U251 cells, respectively. In HUVECs, the most significantly up-regulated pro-inflammatory cytokine/chemokine-related genes (log2FC ≥ 2.25) were 11, including IFI44L, IFIT1, IFI6, IFIT3, BST2, IFIT2, ISG15, NT5E, IFIH1, PLAAT4, and HMGA2. In U251 cells, the most significantly up-regulated pro-inflammatory cytokine/chemokine-related genes (log2FC ≥ 2.25) were 14, including CXCL11, CXCL10, APOL1, PLAAT4, IFI6, NT5E, PTX3, GCH1, C3, CARD16, IRF1, CXCL3, CXCL2, and BIRC3 (Figure 8A, B and Supplement S9). A total of 39 pro-inflammatory cytokine/chemokine-related genes were found to overlap between HUVECs and U251 cells (Figure 8C and D). Furthermore, reactome analysis showed that pro-inflammatory cytokine/chemokine-related genes in HUVECs and U251 cells mainly enriched in cytokine signaling in the immune system (R-HSA-1280215), immune system (R-HSA-168256), interferon signaling (R-HSA-913531), DDX58/IFIH1-mediated induction of interferon-alpha/beta (R-HSA-168928), the innate immune system (R-HSA-168249), ISGF3 binds the ISRE promoter elements in IFN-stimulated genes (R-HSA-1015699), expression of IFN-induced genes (R-HSA-1015702), interferon alpha/beta signaling (R-HSA-909733), and signaling by interleukins (R-HSA-449147) (Figure 8E and Supplements S10, S11). These results help make a possible conclusion that increased miR-103b in TNBC-BM exosomes triggers inflammatory PMN through its regulation on endothelial cells and astrocytes.

**Figure 8.**
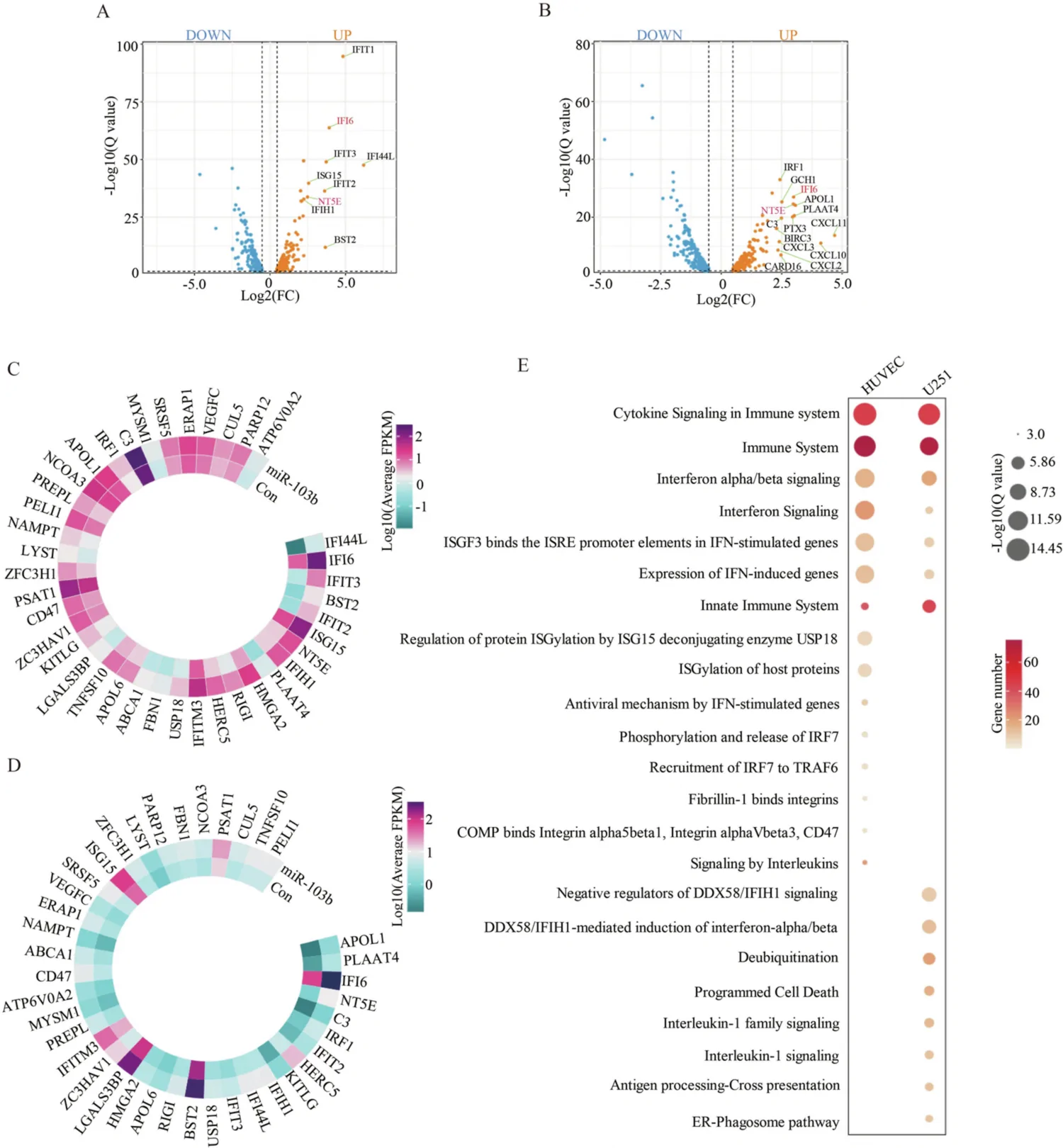
Inflammatory cytokine analysis of miR-103b-regulated DEGs in HUVECs and U251 cells. (A) and (B) Volcano plots showing the distribution of miR-103b-regulated inflammatory cytokines in HUVECs and U251 cells, respectively. Up: up-regulated genes, Down: down-regulated genes. (C) and (D) Circular heat map showing the expression of the overlapped inflammatory cytokines in HUVECs and U251 cells, respectively. (E) Dot plot showing the enriched Reactome pathway of miR-103b-regulated inflammatory cytokines in HUVECs and U251 cells (U251).

### Bioinformatic target analysis of miR-103b targets

The primary mechanism by which miRNAs exert their function is through the destabilization of target mRNAs, leading to degradation and subsequent control of intracellular levels, which represents a major post-transcriptional regulatory pathway.^28-30^ To delineate the direct targets of miR-103b in HUVECs and U251 cells, we performed a comprehensive bioinformatic analysis using four independent algorithms: miRBase V22.1, TargetScan8.0, miRDB, and miRWalk. The results identified 1659 and 2085 differentially expressed genes (DEGs) as potential targets of miR-103b in HUVECs and U251 cells, respectively (Figure 9A and B; Supplementary S12, S13). An intersection analysis revealed 216 downregulated consensus targets (Figure 9C and D). In order to identify the miR-103b-regulated potential hub targets, the degree and eccentricity algorithms were performed using the CytoHubba software plug-in of Cytoscape. As presented in Figure 9E, 33 hub targets, including ACTB, ACTG1, ITGB1, TJP1, CENPA, SDHC, SLC2A1, PGK1, and others, were identified (Figure 9E; Supplementary S14). Furthermore, GO analysis revealed that the hub targets were enriched mainly in biological processes associated with maintenance of the BBB, protein localization to the junction, and the mitotic process (Figure 9F; Supplementary S15). Moreover, Wikipathways analysis demonstrated that hub targets were significantly enriched in pathways associated with glucose metabolism and molecular adhesion (Figure 9G; Supplementary S16).

**Figure 9.**
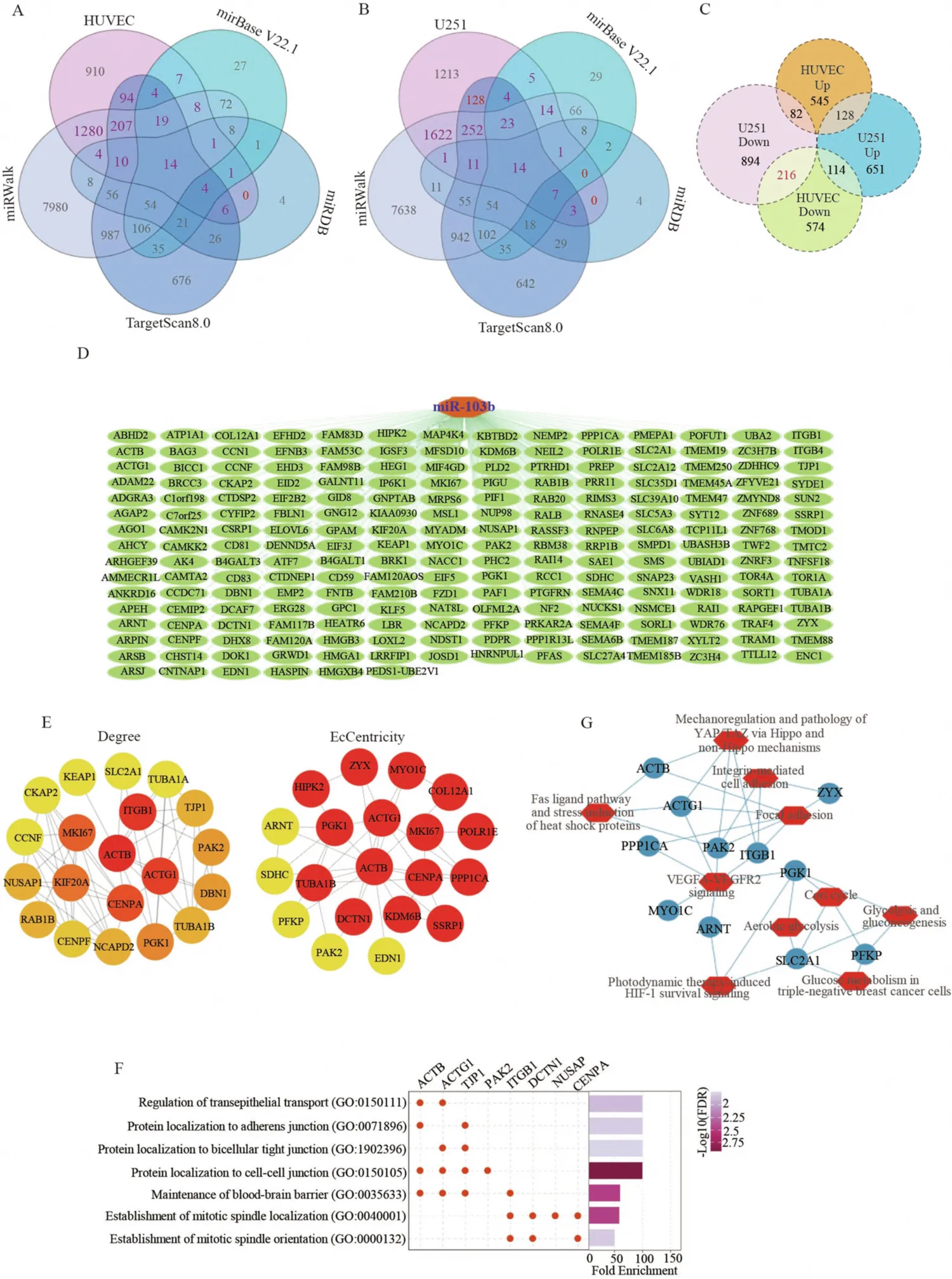
Bioinformatic target analysis of miR-103b-regulated DEGs (A) and (B) Venn diagram showing the overlap of miR-103b-regulated DEGs with predicted target genes of miR-103b from the miRBase database, TargetScan8.0 database, miRDB database, and miRWalk database in HUVECs and U251 cells, respectively. (C) Venn diagram showing the overlap of the predicted target genes of miR-103b in HUVECs and U251 cells (D) Image showing the 216 direct targets of miR-103b. (E) Twenty hub genes and their interaction obtained using Degree and EcCentricity algorithms. (F) Image showing the biological process enrichment of miR-103b-regulated hub targets. (G) Network image showing the major enriched Wikipathway of hub targets.

## Discussion

### miR-103b increased in the exosomes derived from MDA-MB-231Brm cells

miR-103b is a member of the evolutionarily conserved miR-103/107 family, with orthologs present in humans and mice.^31^ miR-103b shares an identical seed sequence with miR-103a and miR-107, the other two members of the miR-103/107 family, suggesting cooperative regulation on target genes and potential functional redundancy.^31^ In our study, exosomal microRNA sequencing demonstrated that miR-103b was significantly up-secreted, whereas miR-103a was significantly down-secreted in the exosomes derived from MDA-MB-231Brm cells compared to those derived from MDA-MB-231cells. Notably, miR-107 was not detected in exosomes from either cell line. Notably, among 14 up-secreted DEMis, miR-103b was not only the most abundant (TPM ≥ 100) but also the most significantly upregulated (|log2FC| ≥ 1). These findings collectively indicate that miR-103b—rather than miR-103a or miR-107—may serve as the primary exosomal miRNA implicated in TNBC-BM pathology. A previous study has indicated that miR-103 and miR-107 were significantly linked to metastasis and poor outcomes in breast cancer.^32^ Sahlberg et al. found that serum miR-103 and miR-107 were elevated in the relapsing TNBC patients, and that increased serum miR-103 was associated with a higher risk of recurrence and a lower OS.^33^ Xiong, B., et al reported that increased miR-103 expression in TNBC tissues showed a significant correlation with aggressive clinicopathological features, including lymph node metastasis (LNM), distant metastasis (DM), and advanced clinical staging. They also observed that miR-103 promotes TNBC cell migration and invasion *in vitro*, suggesting its direct involvement in metastatic progression.^34^ Consistent with these findings, both our exosomal microRNA sequencing and qPCR analyses revealed a significant increase in miR-103b in exosomes derived from MDA-MB-231Brm cells compared to their parental cells. Thus, our data, together with published evidence, converge to nominate the miR-103 family as a key oncogenic driver and a promising biomarker in metastatic TNBC. Taken together, integrating our results with existing evidence, further investigation is warranted to systematically assess the clinical significance of serum exosomal miR-103b in TNBC-BM, particularly its utility as a non-invasive biomarker for diagnosis and disease monitoring.

### miR-103b compromised the tight junction and increased permeability of the BBB

Increased permeability of the BBB is an important characteristic of a PMN for tumor metastasis to the brain. An *in vivo* study has previously shown that miR-103 increases the permeability of the BBB through regulating the expression of caveolin-1 (CAV1) and endothelial tight junctions (TJs).^35^ Similarly, our *in vivo* data revealed that miR-103b-treated mice exhibited obvious FITC-dextran leakage, corroborating the role of the miR-103 family in increasing the permeability of the BBB. Endothelial junction dysfunction is a well-established determinant of increased permeability of the BBB in the pathology of tumor metastasis to the brain.^23^ ^,^ ^36^ ^,^ ^37^ Hepatoma cell-derived exosomal miR-103 was reportedly internalized by vascular endothelial cells and impaired vascular endothelial junction integrity, thereby facilitating transendothelial invasion.^38^ Corroborating these findings, our live-cell microscopy confirmed the uptake of exosomes derived from MAD-MB-231Brm cells by HUVECs. Subsequent staining results of ZO-1 (TJP1) demonstrated the miR-103b-mediated compromise of the TJs in HUVECs. These data collectively suggest the role of TNBC-BM-derived exosomal miR-103b in breaching the endothelial barrier of the BBB, an important characteristic of brain PMN during TNBC-BM.

In the BBB, TJs and adherens junctions (AJs) form the basic endothelial barrier structure. TJs are orchestrated by three integral membrane proteins, including claudin, occludin, and junction adhesion molecules (JAMs), as well as several cytoplasmic accessory proteins, including ZO-1 (TJP1), ZO-2 (TJP2), ZO-3 (TJP3), cingulin (CGN), and others.^39^ In our study, RNA sequencing data showed that miR-103b did not affect the mRNA levels of genes encoding three integral membrane proteins and AJ-related proteins in HUVECs. Cytoplasmic accessory proteins (ZO-1, ZO-2, ZO-3, and cingulin) were identified as essential for tight junction formation by assembling claudins, occludin, and JAMs and anchoring this multimolecular complex to the actin cytoskeleton.^40^ In the current study, RNA sequencing data and qPCR results ruled out that miR-103b significantly downregulated TJP1 and TJP3 in HUVECs and U251 cells. Moreover, TJP1 was predicted to be a direct target of miR-103b, supporting a post-transcriptional regulatory mechanism. These results are similar to previous observations that miR-103 downregulated TJP1 in endothelial TJs.^35^ ^,^ ^38^ In addition to TJ-related cytoplasmic accessory proteins, the actin cytoskeleton is indicated to play a crucial role in maintaining the endothelial junction structure.^41^ A recent study demonstrated that actin polymerization was essential for the elongation of TJs and the subsequent assembly of TJ belts.^42^ Diminished actin filament organization reportedly destroyed the BBB and mediated BCBM.^18^ In the current study, miR-103b-mediated downregulation of ACTB and ACTG1 was observed in both HUVECs and U251 cells. KEGG analysis demonstrated the involvement of ACTB and ACTG1 in the tight junction pathway. Target analysis showed that ACTB and ACTG1 were direct targets of miR-103b. Collectively, our results propose a direct mechanism by which TNBC-BM-derived exosomal miR-103b impairs the endothelial barrier of the BBB via targeting the cytoplasmic accessory gene TJP1 and the actin cytoskeleton genes ACTB and ACTG1.

The basement membrane, consisting of four major ECM proteins: collagen IV, laminin, nidogen, and perlecan, is an important non-cellular component of the BBB.^43^ Astrocytic laminin reportedly regulates pericyte differentiation, and astrocytic laminin-knockdown mice showed patchy and diffuse basement membrane as well as BBB breakdown.^44^ In a study of brain metastasis of breast cancer, an abnormal astrocytic basement membrane is indicated to be involved in increased BBB permeability.^45^ In our study, RNA sequencing data revealed that miR-103b selectively downregulated the expression of LAMB2, LAMB3, and NID1 in U251 cells but not in HUVECs. These results suggest that the dysregulation of astrocytic laminin may be a novel mechanism underlying miR-103b-mediated BBB disruption.

### miR-103b triggers apoptosis of cellular components within the neurovascular unit (NVU)

The impairment of key cellular components within the NVU is implicated in the formation of brain PMN. For instance, an ultrastructure study revealed damaged endothelial cells and progressive astrocyte loss in brain metastatic lesions of non-small cell lung cancer.^46^ TNBC-BM cells are indicated to induce endothelial apoptosis, as evidenced by the presence of cleaved caspase-3-positive endothelial cells during metastatic extravasation in the mouse brain.^47^ Exosomes derived from lung cancer reportedly decrease the cell viability of astrocytes by promoting their apoptosis, which accelerates the formation of PMN.^48^ However, the mechanism underlying the apoptosis of endothelial cells and astrocytes in PMN during TNBC-BM remains elusive. Several studies have revealed that miR-103/107 induces cellular apoptosis by targeting genes in core apoptosis pathways.^49^ ^,^ ^50^ In our study, miR-103b markedly reduced cell viability and promoted apoptosis in HUVECs and U251 cells. Furthermore, GO analysis revealed a significant enrichment of miR-103b-regulated DEGs in apoptosis-associated biological processes, such as regulation of the apoptotic process (GO:0042981) in HUVECs and the intrinsic apoptotic signaling pathway in response to DNA damage by p53 class mediator in U251 cells. Meanwhile, KEGG analysis revealed the enrichment of miR-103b-regulated DEGs in the apoptosis pathway in both HUVEC and U251 cells. Notably, among the genes enriched in the apoptosis pathway, GADD45B and TNFSF10, two well-established pro-apoptosis genes,^51^ ^,^ ^52^ were significantly increased in both HUVEC and U251 cells. Both GADD45B and TNFSF10 are p53 response genes.^53^ ^,^ ^54^ Recently, an in vivo study has revealed that the loss of zinc finger protein 768 (ZNF768), an emerging transcription factor that plays key roles in driving proliferation, amplifies p53 action.^55^ Villot R et al reported that ZNF768 interacts with and represses p53 phosphorylation and activity.^56^ In our study, RNA sequencing data and qPCR results revealed that miR-103b significantly down-regulated the expression of ZNF768 in both HUVECs and U251 cells. Moreover, ZNF768 was predicted to be a direct target of miR-103b. Accordingly, the direct downregulation of ZNF768 by miR-103b and the subsequent activation of p53-mediated pro-apoptotic gene transcription, such as GADD45B and TNFSF10, may be one of the major causes of miR-103b-induced apoptosis in HUVECs and U251 cells.

### miR-103b elevated intracellular ROS of cellular components within the NVU

In addition to triggering apoptosis, our data demonstrated that miR-103b caused mitochondrial dysfunction and ROS accumulation in HUVECs and U251 cells. GO and KEGG analysis showed that miR-103b-regulated DEGs were obviously enriched in oxidative stress-associated biological processes in HUVECs and U251 cells. Succinate dehydrogenase (SDH; also called mitochondrial respiratory chain complex II) is an enzyme that catalyzes the oxidation of succinate into fumarate in the TCA cycle and the subsequent transfer of extracted electrons to ubiquinone via the electron-transport chain. SDHC, a subunit of SDH, provides the binding site for ubiquinones (an element of the electron-transport chain). SDHC knockdown or mutation attenuates SDHC activity and increases the production of ROS signaling.^57^ ^,^ ^58^ RNA sequencing data and qPCR results showed that miR-103b significantly down-regulated the expression of SDHC in both HUVECs and U251 cells. Moreover, SDHC was predicted to be a direct target of miR-103b and also a hub target. Therefore, direct downregulation of SDHC by miR-103b is likely the major triggering event responsible for the abnormal mitochondrial oxidative respiration and increased ROS production observed in HUVECs and U251 cells.

### MiR-103b might be associated with the formation of inflammatory PMN in TNBC-BM pathology

Increased inflammation is suggested as an important characteristic of PMN in TNBC-BM pathology.^59^ ^,^ ^60^ Peritumoral astrocytes reportedly contribute to TNBC progression in the brain through the release of inflammatory cytokines.^61^ Type I interferon (IFN) is indicated as a key mediator in reshaping the overall immune landscape of the brain PMN.^62^ A recent study reported that astrocyte co-culture with TNBC cells leads to a pronounced increase in the expression of type I IFN signaling-associated genes, such as IFI6, IFIT3, IFITM3, IRF1, and ISG15. This activated astrocytic IFN signaling creates a sustained low-level activated IFN microenvironment in brain metastatic lesions, which mediates a pro-metastatic effect through increasing the recruitment of monocytic myeloid cells.^63^ In our study, inflammatory cytokine analysis revealed that miR-103b significantly up-regulated the IFN signaling-associated genes in U251 and HUVECs. Further reactome analysis showed that pro-inflammatory cytokine/chemokine-related genes in HUVECs and U251 cells were enriched mainly in immune- and IFN-associated pathways. These data not only further confirm the effect of TNBC-BM cells on the activation of the astrocyte IFN response but also suggest that increased exosomal miR-103b in TNBC-BM cells may play an important role in the sustained low-level activated IFN microenvironment in the brain PMN. In addition to pro-inflammatory cytokines, chemokines are another major shaper for the immune landscape of the brain PMN. CXCL10 is indicated to foster an immunosuppressive PMN and promote breast brain metastasis.^64^ Doron, H. et al. reported that CXCL10 is upregulated in metastasis-associated astrocytes and facilitates tumor brain tropism via the CXCL10-CXCR3 signaling axis.^65^ In our study, RNA sequencing data demonstrated that miR-103b obviously upregulated the expression of CXCL10, CXCL11, CXCL2, and CXCL3 in U251 cells. Accordingly, increased exosomal miR-103b in TNBC-BM cells may be a key contributor to the formation of inflammatory PMN in TNBC-BM pathology.

### miR-103b might be associated with metabolic alterations of PMN in TNBC-BM pathology

Metabolic alterations within the brain PMN are indicated to facilitate metabolic exchanges between brain-resident and metastatic cells through impacting metastatic fitness and mitigating inflammation, which enables tumor survival, contributing to brain metastasis.^66^ ^,^ ^67^ miR-103/miR-107 is implicated in the regulation of metabolic alterations, such as glucose metabolism and lipid metabolism.^68^ ^,^ ^69^ In line with these findings, our results of KEGG analysis demonstrated the enrichment of miR-103b-regulated DEGs in multiple metabolism-associated pathways in HUVECs and U251 cells, including glycolysis/gluconeogenesis and fatty acid metabolism. The expression of the glucose transporter SLC2A1 (GLUT1) in intratumor microvessels is reportedly negatively correlated with TNBC-BM.^70^ Downregulated SLC2A1 in brain PMN cells, such as astrocytes, reduces their glucose uptake, leading to increased glucose availability for metastatic tumor cells in the brain.^71^ Our RNA sequencing data and qPCR results showed that miR-103b significantly down-regulated the expression of glucose metabolism-associated genes, including SLC2A1, PGK1, and PFKP, in both HUVECs and U251 cells. Target gene analysis showed that SLC2A1, PGK1, and PFKP were predicted to be direct hub targets of miR-103b. Further WikiPathway analysis of the hub targets shows the enrichment of hub targets in glucose metabolism-associated pathways, such as glucose metabolism in triple-negative breast cancer cells, glycolysis and gluconeogenesis, aerobic glycolysis, and the cori cycle. Accordingly, our data implicate that elevated exosomal miR-103b in TNBC-BM cells may be a key driver of metabolic reprogramming of the brain PMN, especially glucose metabolism of intratumoral microvessels and astrocytes.

### Study limitations

Considering that primary human brain microvascular endothelial cells and astrocytes, two key components of the BBB, are technically and ethically challenging to isolate and culture and that significant donor-to-donor variability in phenotype, the in vitro experiments in this study were performed using HUVECs and U251 cells—both immortalized cell lines. This model selection was designed to establish a clear and controllable experimental platform to delineate a framework molecular portrait of the functional effects of miR-103b, defining its core roles in the formation of PMN, such as tight junction regulation, apoptosis, oxidative stress, and inflammatory microenvironment modulation, thereby providing direction for subsequent investigations. However, there are still some limitations. Although immortalized HUVECs have been demonstrated to have a miRNA profile that is the closest correlation with that of BMECs, and they have been extensively used as standard *in vitro* surrogates in numerous blood‒brain barrier and neurovascular unit studies;^23^ ^,^ ^72^ ^,^ ^73^ they are not derived from the brain microvasculature and differ from brain microvascular endothelial cells in terms of tight junction integrity and transporter expression. Similarly, although U251 cells offer preliminary insights into glial lineage responses and several studies have successfully used them to mimic astrocyte‒endothelial cell crosstalk in simplified in vitro BBB models to study intercellular signaling between glial and endothelial compartments,^74^ U251 cells are tumor-derived and do not fully recapitulate the properties of normal primary astrocytes. Furthermore, immortalized cell lines inherently possess incomplete barrier functions, which may lead to underestimation or overestimation of the actual impact of miR-103b on BBB permeability. Therefore, the molecular target and pathway revealed in this study need further systematic validation in more physiologically relevant models, such as human BMECs and primary cultured astrocytes, to gain a more definitive and mechanistic understanding of the pathological significance of exosomal miR-103b in TNBC-BM.

In addition, the core biological function of miRNAs is their ability to directly negatively regulate gene transcription by binding to the 3′-UTRs of target mRNAs and thereby inhibiting target gene expression, mainly at the post-transcriptional mRNA level. In our study, 216 genes were predicted as putative target genes of miR-103b by four independent algorithms: miRBase V22.1, TargetScan8.0, miRDB, and miRWalk. Although mRNA sequencing and qPCR analysis also verified the down-regulation of key target genes such as *TJP1, ACTB, ACTG1, SDHC, and ZNF768* following treatment with the miR-103b mimic in both HUVECs and U251 cells, whether miR-103b directly binds to the 3′-UTRs of these predicted targets to exert its regulatory effects requires further validation, such as dual-luciferase reporter assays and Western blotting.

Finally, while the present study established the PMN-remodeling function of exosomal miR-103b through target gene validation and cellular assays, we acknowledge that the downstream signaling pathways mediating these effects—including but not limited to p53, MAPK, and AMPK cascades—remain to be systematically characterized. Our results of the GO and KEGG enrichment analyses suggested the potential involvement of multiple signaling networks, which warrants further investigation. To address this, further investigation using a multiplex signaling pathway panel are warranted to provide a comprehensive view of the signaling landscape downstream of miR-103b. These future experiments will complement the current findings and further elucidate the mechanistic network underlying miR-103b-mediated PMN remodeling.

## Conclusion

Collectively, our findings provide compelling evidence that elevated miR-103b in exosomes derived from TNBC-BM cells remodels the brain PMN through its regulation of astrocytes and BMECs (Figure 10). These observations offer novel mechanistic insights into the formation of the PMN during TNBC-BM and suggest the potential utility of serum exosomal miR-103b as a diagnostic biomarker for TNBC-BM. Nevertheless, further in vivo studies are needed to clarify its impact on BBB integrity and brain niche inflammation, and clinical cohorts are needed to fully establish its role in PMN formation and biomarker potential.

**Figure 10.**
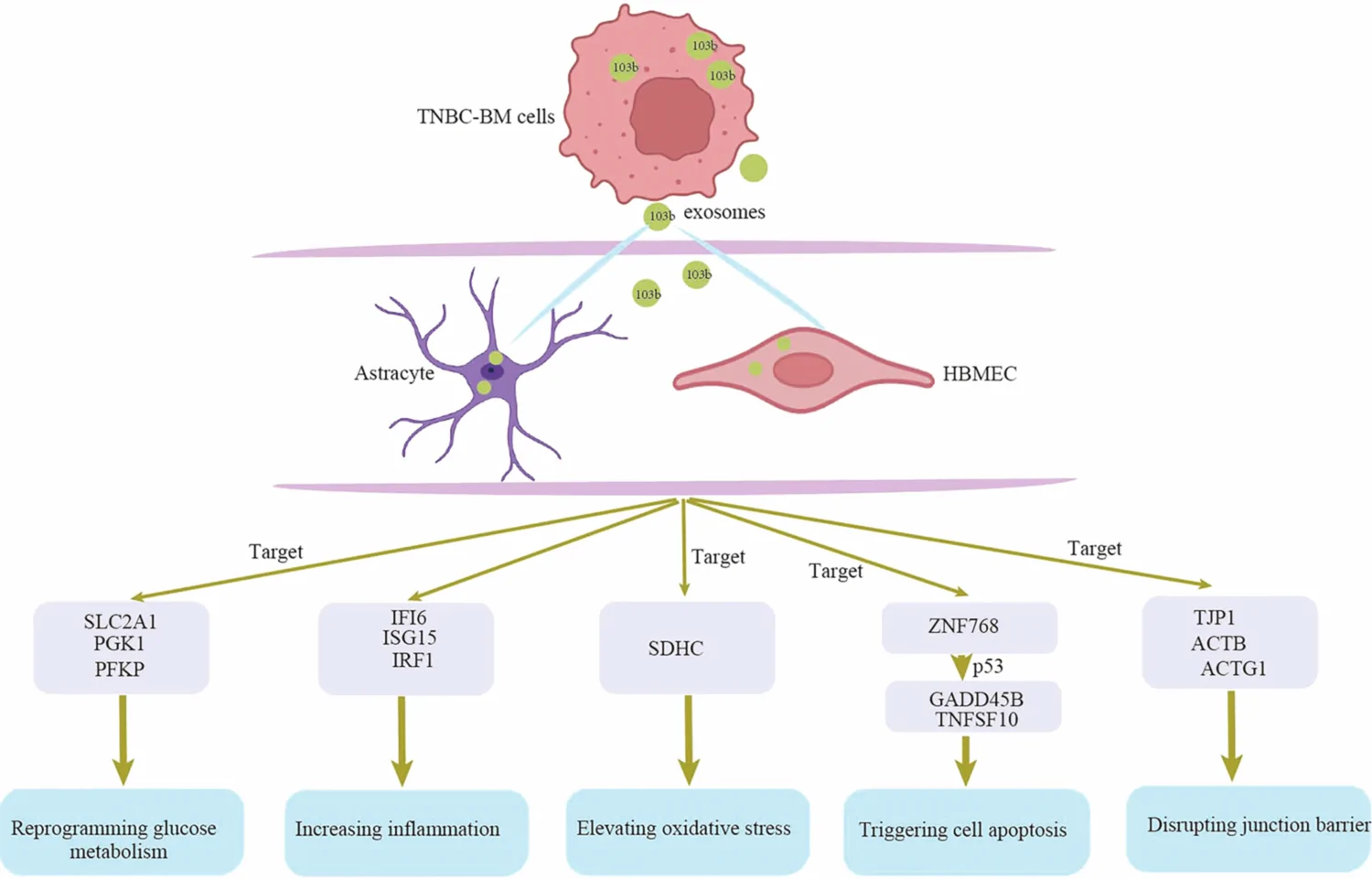
A schematic illustrates the remodeling of elevated miR-103b in exosomes derived from TNBC-BM cells on the brain PMN in TNBC-BM pathology.

## Supplementary Material

Supplementary material Author Checklist.pdf

Supplementary Material Supplementary legend.docx

## Data Availability

The data analyzed for this study are published in this manuscript. Supplementary data are available in the figshare repository (https://doi.org/10.6084/m9.figshare.32204802 and https://doi.org/10.6084/m9.figshare.32103541). In addition, sequencing data have been deposited in the Gene Expression Omnibus (GEO) database under accession number GSE320274. The data will be publicly available upon publication of this manuscript, with no hold period applied.
