# Supplementary material for: Elevated miR-103b in exosomes derived from brain-metastatic triple-negative breast cancer cells remodels the brain pre-metastatic niche (PMN)
Source: Cancer Biol Ther. 2026 Sep 25;27(1):2725357. doi: 10.1080/15384047.2026.2725357 (PMC13618856; doi:10.1080/15384047.2026.2725357)
Supplement: Supplementary file 2 [file KCBT_A_2725357_SM5489.docx]

**Supplementary Files**

**Supplementary S1** live-cell microscopy video of the uptake of exosomes by HUVECs

**Supplementary S2** List of differentially secreted miRNAs in exosomes derived from MDA-MB-231 versus MDA-MB-231Brm cells.

**Supplementary S3** List of miR-103b-regulated differentially expressed genes in HUVECs.

**Supplementary S4** List of miR-103b-regulated differentially expressed genes in U-251 cells.

**Supplementary S5** List of the biological process enrichment of miR-103b-regulated DEGs in HUVECs.

**Supplementary S6** List of the biological process enrichment of miR-103b-regulated DEGs in U-251 cells.

**Supplementary S7** List of the signaling pathway enrichment of miR-103b-regulated DEGs in HUVECs.

**Supplementary S8** List of the KEGG signaling pathway enrichment of miR-103b-regulated DEGs in U-251 cells.

**Supplementary S9** List of the inflammatory cytokines analysis in HUVECs and U-251 cells

**Supplementary S10** List of the Reactome signaling pathway enrichment of miR-103b-regulated inflammatory cytokines in HUVECs.

**Supplementary S11** List of the Reactome signaling pathway enrichment of miR-103b-regulated inflammatory cytokines in U-251 cells.

**Supplementary S12** List of the predicted miR-103b-targeted genes in U-251 cells from the miRBase database, TargetScan8.0 database, miRDB database, and miRWalk database.

**Supplementary S13** List of the predicted miR-103b-targeted genes in HUVECs from the miRBase database, TargetScan8.0 database, miRDB database, and miRWalk database.

**Supplementary S14** List of the hub targets.

**Supplementary S15** List of the biological process enrichment of the hub targets.

**Supplementary S16** List of the Wikipathway enrichment of the hub targets.
